# Using the Framework for Reporting Adaptations and Modifications-Expanded (FRAME) to study lung cancer screening adaptations in the Veterans Health Administration

**DOI:** 10.21203/rs.3.rs-1862731/v1

**Published:** 2022-08-08

**Authors:** Thomas E. Strayer, Lucy B. Spalluto, Abby Burns, Christopher J. Lindsell, Claudia I. Henschke, David F. Yankelevitz, Drew Moghanaki, Robert S. Dittus, Timothy J. Vogus, Carolyn Audet, Sunil Kripalani, Christianne L. Roumie, Jennifer A. Lewis

**Affiliations:** Vanderbilt University Medical Center; Vanderbilt University Medical Center; Veterans Health Administration; Vanderbilt University Medical Center; Mount Sinai Medical Center: Mount Sinai Health System; Mount Sinai Medical Center: Mount Sinai Health System; UCLA Health System: University of California Los Angeles Health System; Vanderbilt University Medical Center; Vanderbilt University; Vanderbilt University Medical Center; Vanderbilt University Medical Center; Vanderbilt University Medical Center

**Keywords:** Frame Framework, Lung cancer screening, Adaptations, Scaled-Implementation, database management, Covid-19

## Abstract

**Background:**

Lung cancer screening includes identification of eligible individuals, shared decision-making inclusive of tobacco cessation, and management of screening results. Adaptations to the implemented processes for lung cancer screening *in situ* are understudied and underreported, with potential loss of important considerations for improved implementation. The Framework for Reporting Adaptations and Modifications-Expanded (FRAME) allows for systematic enumeration of adaptations to implementations of evidence-based practices. We used FRAME to study adaptations in lung cancer screening processes that were implemented as part of a Veterans Health Administration (VHA) Enterprise-Wide Initiative.

**Methods:**

We conducted semi-structured interviews at baseline and 1-year intervals with lung cancer screening program navigators at 10 Veterans Affairs Medical Centers (VAMC) between 2019–2021. Using this data, we developed baseline (1st) process maps for each program. In subsequent years (year 1 and year 2), each program navigator reviewed the process maps. Adaptations in screening processes were identified, recorded and mapped to FRAME categories.

**Results:**

A total of 14 program navigators across 10 VHA lung cancer screening programs participated in 20 interviews. In year 1 (2019–2020), seven programs were operational and of these, three reported adaptations to their screening process that were either planned and in response to COVID-19. In year 2 (2020–2021), all 10 programs were operational. Programs reported 14 adaptations in year 2. These adaptations were both planned and unplanned and often triggered by increased workload; 57% of year 2 adaptations were related to identification and eligibility of Veterans and 43% were related to follow-up with Veterans for screening results. Throughout the 2 years, adaptations related to data management and patient tracking occurred in 6 of 10 programs to improve the data collection and tracking of Veterans in the screening process.

**Conclusions:**

Using FRAME, we found that adaptations occurred throughout the lung cancer screening process but primarily in the areas of patient identification and communication of results. These findings highlight considerations for lung cancer screening implementation and potential areas for future intervention.

## Background

Over the past two decades, implementation science has facilitated the translation of evidence-based practices (EBP) into clinical practice with goals of fidelity, scalability, and effective dissemination.(1) Over 150 implementation frameworks exist to guide the uptake and delivery of EBPs into clinical practice. Once implemented, adaptations are changes in the deployed processes that may have an impact on intervention (effect) outcomes.(2, 3) Adaptations are often overlooked yet should be considered the foundation for translation across health systems.(4, 5) The importance of adaptations can be further considered for two reasons: (1) they highlight the difficulties of maintaining fidelity within real-world clinical settings, and (2) adaptations can help to achieve scalability and sustainability of EBPs across organizations.(6–8) Our aim is to identify and understand adaptations of form in workflow processes within 10 Veterans Affairs (VA) lung cancer screening programs using the Framework for Reporting Adaptations and Modifications-Expanded (FRAME).(4)

### Framework for Reporting Adaptations and Modifications-Expanded (FRAME)

**Figure 1:**
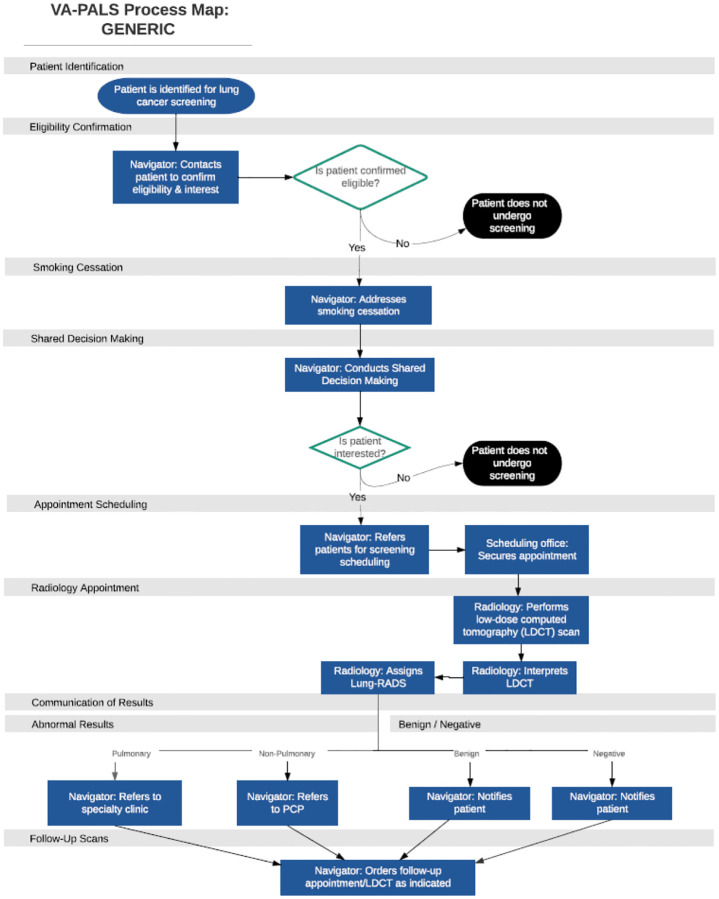
VA-PALS Adapted FRAME

FRAME was created to guide the systematic enumeration of adaptations in implementation. The intent is to ensure sufficient documentation and description of the adaptations that they are informative to those overcoming implementation barriers and developing implementation strategies. FRAME highlights: 1) *when* an adaptation occurs, 2) whether the adaptations were *planned*, 3) *who* decided to make the adaptations, 4) the *adaptation* and 5) the *phase of intervention delivery* in which the adaptation occurred. By reporting at this level of detail, future adopters can evaluate the flexibility of an EBP and the level of detail needed to implement EBPs or address adaptations to the evidence-based practice at their facility or to their particular context.(4) To complete this work, we used FRAME to reflect lung cancer screening process adaptations (i.e. these changes were on the method of delivery of lung cancer screening by lung cancer screening programs). See Fig. 1 for FRAME. For this work, we were focused on documenting the process adaptation in form versus the impact (outcomes) on the function.(9)

### Evidence-based Practice: Lung Cancer Screening

Multiple studies including two large, randomized clinical trials, found that annual lung cancer screening with low-dose CT reduced lung cancer specific mortality in a high-risk population (those who are older and have extensive smoking histories).(10–13) In 2013, the U.S. Preventive Services Task Force (USPSTF) issued a Grade B recommendation for lung cancer screening in individuals who currently or formerly smoked cigarettes (quit within last 15 years) with a 30 pack-year history of cigarette smoking and between the ages of 55 and 80. USPSTF expanded its eligibility criteria in 2021 to a younger population (starting at age 50) with less smoking history (20 pack-years).(14, 15) Individuals should also be asymptomatic and able to undergo curative treatment (surgery or radiation). The screening is covered by the Centers for Medicare and Medicaid Services in a similar population.(16)

### Intervention: Lung Cancer Screening Programs

Centralized lung cancer screening programs have been shown to increase uptake of high-quality lung cancer screening and improve clinical outcomes.(17, 18) VA-Partnership to increase Access to Lung Screening (VA-PALS) is a Veterans Health Administration (VHA) Enterprise-Wide Initiative (EWI) that implemented lung cancer screening programs for high-risk Veterans.(19, 20) Each program provides lung cancer screening with low-dose computed tomography (LDCT).

VA-PALS began in 2017 at 10 Veteran Affairs Medical Centers (VAMCs) with resources that included a program navigator, CT quality control calibration tools, provider training, a support network, and program management software. Each program is led by a site director and was allowed to design their own screening processes based on local resources, environmental contexts, and stakeholder engagement (e.g. primary care, radiology, pulmonology, medical oncology, thoracic surgery).(19) Generalized processes for a VA-PALS lung cancer screening program are outlined in Fig. 2 and can be categorized into the following: 1) Identification and eligibility; 2) Shared decision-making; 3) Smoking cessation services; 4) LDCT with interpretation; 5) Result communication; and 6) Data management/Veteran tracking.

The process begins with identification of eligible Veterans who, if they agree, are referred to the lung cancer screening program. Once referred to the program, a shared decision-making encounter occurs between the program navigator and the Veteran. If the Veteran agrees to screening, an LDCT exam is ordered. Smoking cessation services (education, counseling, and/or medications) are incorporated into the screening process during the shared decision-making encounter for individuals who currently smoke cigarettes. The Veteran undergoes the LDCT screening, and the imaging is reviewed and interpreted by a radiologist. Screening results are sent to the navigator who communicates them to the Veteran. The navigator takes appropriate next steps based on screening results, which can include annual repeat screening if the screening is negative, a short-term interval follow-up exam for indeterminant screenings, or a referral for further evaluation of findings concerning for lung cancer.

For program data management, each program initially used a Microsoft Excel spreadsheet for management and tracking of Veterans throughout the screening process. The VA-PALS software development team deployed the Early Lung Cancer Action Program (ELCAP) software system for use within VHA as an open-source tool called VAPALS-ELCAP Management System. (20, 21) VAPALS-ELCAP facilitates patient tracking, data aggregation, and prospective reporting of the entire lung cancer screening process, from patient identification, shared decision-making, screening results, patient adherence, procedures performed, and cancers diagnosed.

## Methods

### Setting

We evaluated adaptations in lung cancer screening processes within programs established by VA-PALS. In 2017, VA-PALS sites included Atlanta, Chicago-Hines, Cleveland, Denver, Indianapolis, Milwaukee, Nashville, Philadelphia, Phoenix, and St. Louis VAMCs. Each of these VAMCs is a 1A/1B complexity-level facility, with 1A being the most complex and 3 being the least complex. This system takes into consideration patient volume, patient case types, number/type of clinical services, presence/size of residency programs, and research performed.(22–24) Each site hired its program navigator and initiated lung cancer screening at different start dates between 2018 and 2020.

### Study Design and Data Collected

We conducted telephone and virtual interviews with program navigators at the start of their program (baseline n = 10, 2019–2020) and every 12 months thereafter (n = 10, 2020–2021).

At the initial interview with a program navigator, a team member (AB) inquired about the navigator’s demographics, professional training, and start date of screening program activities. At each interview (initial and subsequent interviews), the interviewers (AB/TS) presented the prior process map to each navigator for their review and the navigators did a walk-through of their processes from the beginning to the end of the current screening workflow of their program. Navigators discussed resources available that impact workflow. Each navigator was specifically probed about process adaptations using FRAME. Electronic notes were recorded for all interviews by two study team members [AB/TS]. A study team member (TS/AB) incorporated notes and used Lucidchart♥ to create process maps that outline clinical workflow, the setting, target population, and screening program team members (e.g., program navigators, clinical providers, radiologists.). We sent each program’s process maps to their navigators (after each interview cycle) for their review, editing, and approval. After approved by each navigator, the process map was considered final for that interview cycle.

### Primary Outcome: Adaptation Categorization

We defined adaptations as interval workflow process changes that impacted lung cancer screening delivery (i.e., the process of getting an eligible Veteran to undergo a screening, not the screening itself). (4,25) Two team members [TS, AB] performed content analysis of final process maps using FRAME. A third investigator [JL] independently repeated this categorization. After each coding, the investigators checked for inconsistencies and any disagreement in categorization of each adaptation (< 10% of responses). All disagreement was resolved by discussion until consensus was reached for each adaptation. The adaptations reported are from year 1 (2019–2020) and year 2 (2020–2021).

This program evaluation is approved by VA Central Institutional Review Board (C-IRB E19–05) and the VA Tennessee Valley Healthcare System Research & Development Committee. Committee approval was granted on November 21, 2019. The VA Organizational Assessment Subcommittee approved the study on October 1, 2019, and the VA Office of Labor and Management Relations (national union approval) approved the study on January 27, 2020. The decision to publish was made by the study team.

## Results

We conducted 20 interviews with 14 navigators at 10 VHA lung cancer screening programs between 2019 and 2021. Most navigators were female (85%) and Advanced Practice Providers (85% Nurse Practitioners, 15% registered nurses). There were three adaptations from 2019–2020 and 14 adaptations from 2020–2021, for a total of 17 adaptations across the two years (Table 1).

### Year 1 adaptations (2019–2020).

Seven programs were operational, and three programs were in the process of hiring a program navigator. Four sites reported no adaptations. Three sites reported “top down” adaptations by organizational leadership that were planned, and reactive, due to the COVID-19 pandemic. These included social distancing and reduction of in-person activities for shared decision-making and smoking cessation services. Adaptations included telehealth or virtual encounters for eligibility and shared decision-making portions of the lung cancer screening, but only three programs needed to adapt in this manner; many were flexible to a pandemic-type setting and utilizing means of communication and scheduling that adhered to social distancing guidelines.

### Year 2 adaptations (2020–2021).

All 10 programs were operational, and nine of the 10 programs reported at least one adaptation. There were a total 14 adaptations reported. Eight adaptations (57%) occurred in the patient identification and eligibility confirmation. Six adaptations (43%) were related to result communication and follow-up with Veterans. All adaptations were decided upon by a combination of the screening team including program leadership and/or navigators. Most adaptations (64%) were due to constrained resources (navigator time). Activities such as reaching Veterans on the phone at a time convenient to the Veteran or by mail, to verify eligibility or let them know next steps in the screening process can take considerable time. To alleviate these resource constraints, navigators reported hiring additional personnel, contacting Veterans via different mechanisms (phone to mail or vice versa), or even reducing sites of recruitment. These adaptations were unplanned and “internal decisions” made between the site director(s) and the navigator in 2021.

### Data Management and Veteran Tracking Adaptations.

All programs began with an Excel spreadsheet, and six of the ten (60%) noted adaptations in their data management system (Table 2). One program began using the VAPALS-ELCAP Management System (10%), 3 (30%) programs used the VA National Lung Cancer Screening Platform (previously known as VISN 23 management system), 1 (10%) program used a REDCAP variation, and 1 (10%) program used LungView©. Reported reasons for adaptation and for not adopting the proposed adaptation included navigator time constraints to enter data and improved tracking of Veterans.

## Discussion

We report adaptations in most stages of the lung cancer screening process, most frequently in the identification and eligibility of individuals for screening; identifying eligible individuals requires obtaining and parsing complex information on smoking history such as start and stop dates and number of packs smoked per day. There were also adaptations in shared decision-making, tobacco cessation, follow up of screening results, and communication of results to patients.

Multiple factors influenced adaptations. Adaptations in year 1 focused on COVID-19 to limit in-person screening-related activities. Adaptations in year 2 were largely focused on time and resource constraints of the navigators. Navigators’ adaptations focused much of their time on recruitment and verification of screening eligibility, as well as communication of results back to Veterans. Adaptations in these activities focused on creating more efficient processes to reach Veterans. Interestingly, there was site level variability in how programs approached constraints on navigator time. Some programs reduced the number of Veterans that were recruited for screening by reducing the number of recruitment settings, while other programs reduced points of contact (using phone calls versus mailed letters, etc.). Only one site adapted to the increasing volume by adding sufficient navigator resources to meet demand (e.g., additional support staff,).

There was variability in available resources and in programs’ approaches to overcome barriers. At some sites, multiple navigators were able to work as a team to coordinate screening activities, while others needed additional support personnel to meet screening demand. One program noted a navigator was on-leave and an outside staff member covered screening program activities. To make communication of results to Veterans more efficient, two programs replaced telephone calls with mailed letters, while another program did the opposite. These adaptations highlight how agile programs were at adjusting workflows to fulfill their commitment and responsibilities.

### Documenting and understanding

All programs had planned adaptations to the data management tools. Part of the VA-PALS program was the development and installation of the VAPALS-ELCAP Management System as a tool for all sites; Microsoft Excel was meant to be only a temporary tool. Barriers to software installation led to a delayed timeline, and only one program was able to use the software system during the study time period. VAPALS-ELCAP has been pilot tested and is currently managing over 1,500 Veterans in a centralized screening program. Since the time of data collection for this study, the tool has been installed at a second program. Many programs adapted to a data management tool used by other VAMCs (National Lung Cancer Screening Care Platform Tool 1.0 (previously the VISN 23 system). All adapting programs cited efficiency as a reason for adaptation. A target of future work may aim to address the step-by-step decision-making pathway for data management tools for future implementation.

## Implications

Methods for implementing evidence-based practices are often adapted, but how and why remains unexplored, especially in nationally scaled interventions.(5) The results of this work highlight variations in lung cancer screening delivery and adaptations to overcome implementation barriers from a national perspective, often highlighting resource constraints as a reason for adaptations, particularly program navigator time. The results also demonstrate that local process variation and adaptations persist even in highly integrated systems. Understanding the complexity of widely scaled pragmatic interventions should support the need for developers to consider and promote adaptations in implementation trials. In addition, these data show the complexity of capturing adaptations and the utility of an established framework such as FRAME.

Understanding adaptations to implementation of evidence-based practices allows for more successful implementation through a better understanding of sites’ unique contextual factors. Much of the existing literature has focused on adaptations to the evidence-based practice to local context.(26–29) For example, many studies discuss adaptations of interventions to fit their unique healthcare systems, clinical practice, and patient population.(26–29) Our work does not focus on the evidence-based practice, LDCT for lung cancer screening (a radiology examination). This work is unique in that the focus is on adaptations in clinical processes using FRAME. Accordingly, we highlight the unique constraints and needs of various sites when implementing the same evidence-based practice across 10 VAMCs. Each site was given the opportunity to learn from practice evolution, which is a core component of identifying implementation strategies for broader adoption. These findings highlight that implementation of evidence-based practices is not static and processes where adaptations were common (identifying eligible patients and communication of screening results to patients) may be key to scalability and sustainability over time.

## Limitations

This study has several limitations. We are unable to assess adaptations completed prior to 2019 or for programs that were not fully operational in year one. Additionally, adaptation reporting relied on program navigators. Reporting bias may exist as programs may both under-report adaptations and over-report adaptations. For each assessed adaptation, we are unable to assess fidelity to the adaptation or the clinical impact of these adaptations on screening outcomes. Furthermore, we evaluated adaptations from process maps reviewed by the navigator at each program; other perspectives were not obtained. There may have been an adaptation to the screening processes of which navigators were unaware. Finally, this study focused on the VHA healthcare system and may not be generalizable to other healthcare systems.

## Conclusion

Using FRAME, we found that most programs made adaptations to components of the lung cancer screening process. Programs also made several adaptations in their lung cancer screening management system to improve efficiency of patient tracking, but what efficiency entailed meant something different to each program. These adaptations highlight challenges encountered and may inform current, and future, lung cancer screening program implementation, especially scalability and sustainability. Future directions include determination of the impact adaptations have on the effectiveness of lung cancer screening programs, namely core functions such as recruitment, shared decision-making, and follow-up.

## Figures and Tables

**Figure 2:** Generic Screening Process Map

**Table 1:** Adaptations by Program Site and Year Utilizing FRAME Framework

Location	Year	Modification Planned?	Who made the decision to modify?	Modification Goal	What process was modified?	At what level of delivery?	Nature of modification	Reasons for adaptations
Site A	2020	Planned and Reactive	Executive Leadership	Meet COVID-19^a^ Precautions	Shared Decision-Making and Smoking Cessation	Patient and Practitioner (program navigator)	Substituting	Organization/Setting Competing Demands and Mandates
	2021	Unplanned	Program Site Director and Specialty Clinician (Pulmonary Chief)	Improve Feasibility or Efficiency	Patient Identification and Eligibility Confirmation	Individual Practitioner (program navigator)	Tailoring/Refining	Provider Resources (time)
Site B^b^	2020	Not operationa						
	2021	Unplanned	Program Navigator and Program Site Directors	Increase, Decrease Reach or Engagement	Patient Identification and Eligibility Confirmation	Patient and Practitioner (program navigator)	Tailoring/Refining	Resources (technology)
	2021	Unplanned	Program Navigator and Program Site Directors	Feasibility	Communication of Results	Patient and Practitioner (program navigator)	Removing Elements	Provider Resources (time)
Site C	2020	No Reported Changes						
	2021	Unplanned	Screening Program Team	Decrease Reach or Engagement	Patient Identification and Eligibility Confirmation	Patient and Practitioner (program navigator)	Removing Elements	Sociopolitical (Funding or Resource Allocation/Availability) Provider Resources (time)
Site D	2020	Planned and Reactive	Executive Leadership	Meet COVID-19 Precautions	Shared Decision-Making and Smoking Cessation	Patient and Practitioner (program navigator)	Substituting	Organization/Setting Competing Demands and Mandates
	2021	Planned and Reactive	Screening Program Team	Improve Feasibility or Efficiency	Patient identification and Eligibility confirmation	Patient and Practitioner (program navigator) and	Shortening/Condensing	Provider Resources (time)
						Clinic Unit Level		
Site E^b^	2020	Not operationa						
	2021	Planned and Reactive	Program Navigator and Program Site Directors	Increase Engagement	Screening and Eligibility Confirmation	Patient and Practitioner (program navigator) and Clinic Unit Level	Adding Elements	Organization/Setting (Location/accessibility)
	2021	Planned and Reactive	Program Navigator and Program Site Directors	Increase engagement (Primary Care)	Communication of Results	Patient and Practitioner (program navigator) | and Clinic Unit Level	Removing Elements	Provider preferences
Site F	2020	No Reported Changes						
	2021	Planned and Reactive	Screening Program Team	Increase Engagement	Patient Identification	Patient and Practitioner (program navigator) and Clinic Unit Level	Adding Elements	Organization/Setting (location/accessibility)
	2021	Planned and Reactive	Screening Program Team	Improve Feasibility or Efficiency	Communication of Results	Patient and Practitioner (program navigator)	Substituting	Provider Resources (time)
Site G	2020	Planned and Reactive	Executive Leadership	COVID-19 Precautions	Shared Decision-Making and Smoking Cessation	Patient and Practitioner (program navigator)	Substituting	Organization/Setting Competing Demands and Mandates
	2021	Unplanned	Navigator	Improve Feasibility or Efficiency	Communication of Results	Patient and Practitioner (program navigator)	Substituting	Provider resources (time)
Site H^b^	2020	Not operationa						
	2021	Planned and Reactive	Screening Program Team	Improve Feasibility or Efficiency	Follow up of screening results	Patient and Practitioner (program navigator)	Tailoring/Refìning	Provider Resources (time)
Site I	2020	No Reported Changes						
	2021	No Reported Changes						
Site J	2020	No Reported Changes						
	2021	Unplanned	Navigator	Improve Feasibility or Efficiency	Patient Identification, Eligibility Confirmation	Patient	Substituting	Provider Resources (time)
	2021	Planned and Proactive	Research Stakeholder and Collaboration Group	Improve Feasibility or Efficiency	Communication of Results	Patient and Practitioner (program navigator)	Tailoring/Refìning	Organization/Setting Competing Demands and Mandates
	2021	Planned	Navigator	Improve Feasibility or Efficiency	Follow-up	Patient	Tailoring/Refìning	Provider Resources (time)

a. COVID-19: coronavirus disease of 2019; EMR: electronic medical record

b. N/A: non-applicable. These programs were not operational during the reporting time period. Cleveland and St. Louis had plans for their workflows, but thes Chicago-Hines was without a navigator at the time of the initial adaptations reporting period. However, the screening program was on-going.

c. The Veteran Affairs Corporate Data Warehouse is the national database comprised of data obtained from the electronic health record system.

**Table 2:** Data Management Adaptations by Site

Location	Baseline Data Tool	Year Change Occurred	New Data Tool	Who made the decision to use new Tool?	Goal of using new Data Tool	Modification Description
Site A	Microsoft Excel	2021	LungView©	Program Directors and Navigators	Improve tracking of Veterans longitudinally	Instead of Microsoft Excel, LungView© was used for its ability to communicate with Veteran electronic medical record and improved ease in tracking.
Site B	Microsoft Excel	2021	VISN 23 System (aka National Lung cancer Screening Care Platform Tool 1.0)	Program Directors and Navigators	To decrease navigator time on data entry	Instead of Microsoft Excel, the National Lung Cancer Screening Care Platform was adopted as it has the capabilities to reduce time of navigator data entry.
Site C	Microsoft Excel	No Reported Changes				
Site D	Microsoft Excel	No Reported Changes				
Site E	Microsoft Excel	No Reported Changes				
Site F	Microsoft Excel	2020	VISN 23 System (National Lung cancer Screening Care Platform Tool 1.0)	Program Directors and Navigators	Improve tracking of Veterans and screenings	Instead of Microsoft Excel, the National Lung Cancer Screening Care Platform was adopted as it has the capabilities to integrate with the electronic health record and improve collaborations with other care team members.
Site G	Microsoft Excel	2020	REDCap System	Program Directors and Navigators	Improve tracking of Veterans longitudinally	This modification took approximately 3-months in 2020 to implement from Microsoft Excel to the VA REDCap system. The overall purpose of this change was to improve the longitudinal tracking of Veteran data.
Site H	Microsoft Excel	No Reported Changes				
Site I	Microsoft Excel	2021	VAPALS-ELCAP Management System	Program Directors and Navigators	Improve tracking of Veterans and screenings	Modification was introduced during 2021. VAPALS-ELCAP is a software system designed for use in the VA for lung cancer screening. Programming is noted to be adaptable to current needs of navigators.
Site J	Microsoft Excel	2021	VISN 23 System and Excel spreadsheet (National Lung cancer Screening Care Platform Tool 1.0)	Program Directors and Navigators	Improve tracking of Veterans longitudinally	In combination with Microsoft Excel, the National Lung Cancer Screening Care Platform was adopted as it has the capabilities to reduce time of navigator and allow for better tracking of Veterans and reduce loss of follow-up.

## Data Availability

All relevant data are within the manuscript and its supporting information files. The datasets used and/or analyzed during the current study are available from the corresponding author on reasonable request. This includes initial and updated process maps for each site location.

## Abbreviations

FRAME: Framework for Reporting Adaptations and Modifications-Expanded
VA: Veteran Affairs
VA-PALS: VA-Partnership to increase Access to Lung Screening
VHA: Veterans Health Administration
VAMC: Veterans Affairs Medical Centers
EBP: evidence-based practices
USPSTF: U.S. Preventive Services Task Force
EWI: Enterprise-Wide Initiative
LDCT: low-dose computed tomography
ELCAP: Early Lung Cancer Action Program
